# Supplementary material for: Asynchronous magnetic resonance elastography: Shear wave speed reconstruction using noise correlation of incoherent waves
Source: Magn Reson Med. 2022 Oct 27;89(3):990–1001. doi: 10.1002/mrm.29502 (PMC9792433; doi:10.1002/mrm.29502)
Supplement: Supplementary file 1 — DATA S1 MATLAB code used to generate the numerical simulations of Figure 1 [file MRM-89-990-s002.zip › k-Wave/helpfiles/kspaceFirstOrder1D.html]

kspaceFirstOrder1D :: Functions (k-Wave)


# kspaceFirstOrder1D

1D time-domain simulation of wave propagation.

## Syntax

```
sensor_data = kspaceFirstOrder1D(kgrid, medium, source, sensor)
sensor_data = kspaceFirstOrder1D(kgrid, medium, source, sensor, ...)
```

## Description

`kspaceFirstOrder1D` simulates the time-domain propagation of compressional waves through a one-dimensional homogeneous or heterogeneous acoustic medium given four input structures: `kgrid`, `medium`, `source`, and `sensor`. The computation is based on a first-order k-space model which accounts for power law absorption and a heterogeneous sound speed and density. If `medium.BonA` is specified, cumulative nonlinear effects are also modelled. At each time-step (defined by `kgrid.dt` and `kgrid.Nt` or `kgrid.t_array`), the acoustic field parameters at the positions defined by `sensor.mask` are recorded and stored. If `kgrid.t_array` is set to `'auto'`, this array is automatically generated using the `makeTime` method of the `kWaveGrid` class. An anisotropic absorbing boundary layer called a perfectly matched layer (PML) is implemented to prevent waves that leave one side of the domain being reintroduced from the opposite side (a consequence of using the FFT to compute the spatial derivatives in the wave equation). This allows infinite domain simulations to be computed using small computational grids.

For a homogeneous medium the formulation is exact and the time-steps are only limited by the effectiveness of the perfectly matched layer. For a heterogeneous medium, the solution represents a leap-frog pseudospectral method with a k-space correction that improves the accuracy of computing the temporal derivatives. This allows larger time-steps to be taken for the same level of accuracy compared to conventional pseudospectral time-domain methods. The computational grids are staggered both spatially and temporally.

An initial pressure distribution can be specified by assigning a matrix (the same size as the computational grid) of arbitrary numeric values to `source.p0`. A time varying pressure source can similarly be specified by assigning a binary matrix (i.e., a matrix of 1's and 0's with the same dimensions as the computational grid) to `source.p_mask` where the 1's represent the grid points that form part of the source. The time varying input signals are then assigned to `source.p`. This can be a single time series (in which case it is applied to all source elements), or a matrix of time series following the source elements using MATLAB's standard column-wise linear matrix index ordering. A time varying velocity source can be specified in an analogous fashion, where the source location is specified by `source.u_mask`, and the time varying input velocity is assigned to `source.ux`.

The field values are returned as arrays of time series at the sensor locations defined by `sensor.mask`. This can be defined in three different ways. (1) As a binary matrix (i.e., a matrix of 1's and 0's with the same dimensions as the computational grid) representing the grid points within the computational grid that will collect the data. (2) As the grid coordinates of two opposing ends of a line in the form [x1; x2]. This is equivalent to using a binary sensor mask covering the same region, however, the output is indexed differently as discussed below. (3) As a series of Cartesian coordinates within the grid which specify the location of the pressure values stored at each time step. If the Cartesian coordinates don't exactly match the coordinates of a grid point, the output values are calculated via interpolation. The Cartesian points must be given as a 1 by N matrix corresponding to the x positions, where the Cartesian origin is assumed to be in the center of the grid. If no output is required, the `sensor` input can be replaced with an empty array `[]`.

If `sensor.mask` is given as a set of Cartesian coordinates, the computed `sensor_data` is returned in the same order. If `sensor.mask` is given as a binary matrix, `sensor_data` is returned using MATLAB's standard column-wise linear matrix index ordering. In both cases, the recorded data is indexed as `sensor_data(sensor_point_index, time_index)`. For a binary sensor mask, the field values at a particular time can be restored to the sensor positions within the computation grid using `unmaskSensorData`. If `sensor.mask` is given as a list of opposing ends of a line, the recorded data is indexed as `sensor_data(line_index).p(x_index, time_index)`, where `x_index` corresponds to the grid index within the line, and `line_index` corresponds to the number of lines if more than one is specified.

By default, the recorded acoustic pressure field is passed directly to the output `sensor_data`. However, other acoustic parameters can also be recorded by setting `sensor.record` to a cell array of the form `{'p', 'u', 'p_max', ...}`. For example, both the particle velocity and the acoustic pressure can be returned by setting `sensor.record = {'p', 'u'}`. If `sensor.record` is given, the output `sensor_data` is returned as a structure with the different outputs appended as structure fields. For example, if `sensor.record = {'p', 'p_final', 'p_max', 'u'}`, the output would contain fields `sensor_data.p`, `sensor_data.p_final`, `sensor_data.p_max`, and `sensor_data.ux`. Most of the output parameters are recorded at the given sensor positions and are indexed as `sensor_data.field(sensor_point_index, time_index)` or `sensor_data(line_index).field(x_index, time_index)` if using a sensor mask defined as opposing ends of a line. The exceptions are the averaged quantities (`'p_max'`, `'p_rms'`, `'u_max'`, `'p_rms'`, `'I_avg'`), the 'all' quantities (`'p_max_all'`, `'p_min_all'`, `'u_max_all'`, `'u_min_all'`), and the final quantities (`'p_final'`, `'u_final'`). The averaged quantities are indexed as `sensor_data.p_max(sensor_point_index)` or `sensor_data(line_index).p_max(x_index)` if using line ends, while the final and 'all' quantities are returned over the entire grid and are always indexed as `sensor_data.p_final(nx)`, regardless of the type of sensor mask.

`kspaceFirstOrder1D` may also be used for time reversal image reconstruction by assigning the time varying pressure recorded over an arbitrary sensor surface to the input field `sensor.time_reversal_boundary_data`. This data is then enforced in time reversed order as a time varying Dirichlet boundary condition over the sensor surface given by `sensor.mask`. The boundary data must be indexed as `sensor.time_reversal_boundary_data(sensor_point_index, time_index)`. If `sensor.mask` is given as a set of Cartesian coordinates, the boundary data must be given in the same order. An equivalent binary sensor mask (computed using nearest neighbour interpolation) is then used to place the pressure values into the computational grid at each time step. If `sensor.mask` is given as a binary matrix of sensor points, the boundary data must be ordered using MATLAB's standard column-wise linear matrix indexing. If no additional inputs are required, the `source` input can be replaced with an empty array `[]`.

Acoustic attenuation compensation can also be included during time reversal image reconstruction by assigning the absorption parameters `medium.alpha_coeff` and `medium.alpha_power` and reversing the sign of the absorption term by setting `medium.alpha_sign = [-1, 1]`. This forces the propagating waves to grow according to the absorption parameters instead of decay. The reconstruction should then be regularised by assigning a filter to `medium.alpha_filter` (this can be created using `getAlphaFilter`).

Note: To run a simple photoacoustic image reconstruction example using time reversal (that commits the 'inverse crime' of using the same numerical parameters and model for data simulation and image reconstruction), the `sensor_data` returned from a k-Wave simulation can be passed directly to `sensor.time_reversal_boundary_data` with the input fields `source.p0` and `source.p` removed or set to zero.

## Inputs

The minimum fields that must be assigned to run an initial value problem (for example, a photoacoustic forward simulation) are marked with a \*.

|  |  |
| --- | --- |
| `kgrid*` | k-Wave grid object returned by `kWaveGrid` containing Cartesian and k-space grid fields |
| `kgrid.t_array*` | evenly spaced array of time values [s] (set to `'auto'` by `kWaveGrid`) |
|  |  |
| `medium.sound_speed*` | sound speed distribution within the acoustic medium [m/s] |
| `medium.sound_speed_ref` | reference sound speed used within the k-space operator (phase correction term) [m/s] |
| `medium.density*` | density distribution within the acoustic medium [kg/m^3] |
| `medium.BonA` | parameter of nonlinearity |
| `medium.alpha_power` | power law absorption exponent |
| `medium.alpha_coeff` | power law absorption coefficient [dB/(MHz^y cm)] |
| `medium.alpha_mode` | optional input to force either the absorption or dispersion terms in the equation of state to be excluded; valid inputs are `'no_absorption'` or `'no_dispersion'` |
| `medium.alpha_filter` | frequency domain filter applied to the absorption and dispersion terms in the equation of state |
| `medium.alpha_sign` | two element array used to control the sign of absorption and dispersion terms in the equation of state |
|  |  |
| `source.p0*` | initial pressure within the acoustic medium |
| `source.p` | time varying pressure at each of the source positions given by `source.p_mask` |
| `source.p_mask` | binary matrix specifying the positions of the time varying pressure source distribution |
| `source.p_mode` | optional input to control whether the input pressure is injected as a mass source or enforced as a dirichlet boundary condition; valid inputs are `'additive'` (the default) or `'dirichlet'` |
| `source.ux` | time varying particle velocity in the x-direction at each of the source positions given by `source.u_mask` |
| `source.u_mask` | binary matrix specifying the positions of the time varying particle velocity distribution |
| `source.u_mode` | optional input to control whether the input velocity is applied as a force source or enforced as a dirichlet boundary condition; valid inputs are `'additive'` (the default) or `'dirichlet'` |
|  |  |
| `sensor.mask*` | binary matrix or a set of Cartesian points where the pressure is recorded at each time-step |
| `sensor.record` | cell array of the acoustic parameters to record in the form `sensor.record = {'p', 'u', ...}`; valid inputs are:    `'p'` (acoustic pressure)    `'p_max'` (maximum pressure)    `'p_min'` (minimum pressure)    `'p_rms'` (RMS pressure)    `'p_final'` (final pressure field at all grid points)    `'p_max_all'` (maximum pressure at all grid points)    `'p_min_all'` (minimum pressure at all grid points)    `'u'` (particle velocity)    `'u_max'` (maximum particle velocity)    `'u_min'` (minimum particle velocity)    `'u_rms'` (RMS particle velocity)    `'u_final'` (final particle velocity field at all grid points)    `'u_max_all'` (maximum particle velocity at all grid points)    `'u_min_all'` (minimum particle velocity at all grid points)    `'u_non_staggered'` (particle velocity on non-staggered grid)    `'I'` (time varying acoustic intensity)    `'I_avg'` (average acoustic intensity) |
| `sensor.record_start_index` | time index at which the sensor should start recording the data specified by `sensor.record` (default = 1) |
| `sensor.time_reversal_boundary_data` | time varying pressure enforced as a Dirichlet boundary condition over `sensor.mask` |
| `sensor.frequency_response` | two element array specifying the center frequency and percentage bandwidth of a frequency domain Gaussian filter applied to the `sensor_data` |

**Note**  For a heterogeneous medium, `medium.sound_speed` and `medium.density` must be given in matrix form with the same dimensions as `kgrid`. For a homogeneous medium, these can be given as scalar values. If the medium is homogeneous and velocity inputs or outputs are not required, it is not necessary to specify `medium.density`.

## Optional Inputs

Optional 'string', value pairs that may be used to modify the default computational settings.

| Input | Valid Settings | Default | Description |
| --- | --- | --- | --- |
| `'CartInterp'` | `'linear'` `'nearest'` | `'linear'` | Interpolation mode used to extract the pressure when a Cartesian sensor mask is given. If set to `'nearest'` and more than one Cartesian point maps to the same grid point, duplicated data points are discarded and `sensor_data` will be returned with less points than that specified by `sensor.mask`. |
| `'CreateLog'` | *(Boolean scalar)* | `false` | Boolean controlling whether the command line output is saved using the diary function with a date and time stamped filename. |
| `'DataCast'` | *(string of data type)* | `'off'` | String input of the data type that variables are cast to before computation. For example, setting to `'single'` will speed up the computation time (due to the improved efficiency of `fftn` and `ifftn` for this data type) at the expense of a loss in precision. This variable is also useful for utilising GPU parallelisation through libraries such as the Parallel Computing Toolbox by setting `'DataCast'` to `'gpuArray-single'`. |
| `'DataRecast'` | *(Boolean scalar)* | `false` | Boolean controlling whether the output data is cast back to double precision. If set to false, `sensor_data` will be returned in the data format set using the `'DataCast'` option. |
| `'DisplayMask'` | *(binary matrix)* or | `sensor.mask` | Binary matrix overlaid onto the animated simulation display. Elements set to 1 within the display mask are set to black within the display. |
| `'LogScale'` | *(Boolean scalar)* or *(numeric scalar)* | `false` | Boolean controlling whether the pressure field is log compressed before display. The data is compressed by scaling both the positive and negative values between 0 and 1 (truncating the data to the given plot scale), adding a scalar value (compression factor) and then using the corresponding portion of a log10 plot for the compression (the negative parts are remapped to be negative thus the default color scale will appear unchanged). The amount of compression can be controlled by adjusting the compression factor which can be given in place of the Boolean input. The closer the compression factor is to zero, the steeper the corresponding part of the log10 plot used, and the greater the compression (the default compression factor is 0.02). |
| `'MovieArgs'` | *(string cell array)* | `{}` | Settings for `VideoWriter`. Parameters must be given as {'param', value, ...} pairs within a cell array (default = {}), where 'param' corresponds to a writable property of a VideoWriter object. |
| `'MovieName'` | *(string)* | `'date-time-kspaceFirstOrder1D'` | Name of the movie produced when `'RecordMovie'` is set to `true`. |
| `'MovieProfile'` | *(string)* | `'Uncompressed AVI'` | Profile input passed to `VideoWriter`. |
| `'PlotFreq'` | *(integer numeric scalar)* | `10` | The number of iterations which must pass before the simulation plot is updated. |
| `'PlotLayout'` | *(Boolean scalar)* | `false` | Boolean controlling whether a four panel plot of the initial simulation layout is produced (initial pressure, sensor mask, sound speed, density). |
| `'PlotPML'` | *(Boolean scalar)* | `true` | Boolean controlling whether the perfectly matched layer is shown in the simulation plots. If set to `false`, the PML is not displayed. |
| `'PlotScale'` | *(numeric two element vector)* or `'auto'` | `[-1, 1]` | [min, max] values used to control the scaling for `imagesc` (visualisation). If set to `'auto'`, a symmetric plot scale is chosen automatically for each plot frame. |
| `'PlotSim'` | *(Boolean scalar)* | `true` | Boolean controlling whether the simulation iterations are progressively plotted. |
| `'PMLAlpha'` | *(numeric scalar or three element vector)* | `2` | Absorption within the perfectly matched layer in Nepers per grid point. |
| `'PMLInside'` | *(Boolean scalar)* | `true` | Boolean controlling whether the perfectly matched layer is inside or outside the grid. If set to `false`, the input grids are enlarged by `PMLSize` before running the simulation. |
| `'PMLSize'` | *(integer numeric scalar or three element vector)* | `10` | Size of the perfectly matched layer in grid points. To remove the PML, set the appropriate `PMLAlpha` to zero rather than forcing the PML to be of zero size. |
| `'RecordMovie'` | *(Boolean scalar)* | `false` | Boolean controlling whether the displayed image frames are captured and stored as a movie using `VideoWriter`. |
| `'Smooth'` | *(Boolean scalar or three element vector)* | `[true, false, false]` | Boolean controlling whether `source.p0`, `medium.sound_speed`, and `medium.density` are smoothed using `smooth` before computation. `'Smooth'` can either be given as a single Boolean value or as a 3 element array to control the smoothing of `source.p0`, `medium.sound_speed`, and `medium.density`, independently. |

## Outputs

If `sensor.record` is not defined by the user:

|  |  |
| --- | --- |
| `sensor_data` | time varying pressure recorded at the sensor positions given by `sensor.mask` |

If `sensor.record` is defined by the user:

|  |  |
| --- | --- |
| `sensor_data.p` | time varying pressure recorded at the sensor positions given by `sensor.mask` (returned if `'p'` is set) |
| `sensor_data.p_max` | maximum pressure recorded at the sensor positions given by `sensor.mask` (returned if `'p_max'` is set) |
| `sensor_data.p_min` | minimum pressure recorded at the sensor positions given by `sensor.mask` (returned if `'p_min'` is set) |
| `sensor_data.p_rms` | rms of the time varying pressure recorded at the sensor positions given by `sensor.mask` (returned if `'p_rms'` is set) |
| `sensor_data.p_final` | final pressure field at all grid points within the domain (returned if `'p_final'` is set) |
| `sensor_data.p_max_all` | maximum pressure recorded at all grid points within the domain (returned if `'p_max_all'` is set) |
| `sensor_data.p_min_all` | minimum pressure recorded at all grid points within the domain (returned if `'p_min_all'` is set) |
| `sensor_data.ux` | time varying particle velocity in the x-direction recorded at the sensor positions given by `sensor.mask` (returned if `'u'` is set) |
| `sensor_data.ux_max` | maximum particle velocity in the x-direction recorded at the sensor positions given by `sensor.mask` (returned if `'u_max'` is set) |
| `sensor_data.ux_min` | minimum particle velocity in the x-direction recorded at the sensor positions given by `sensor.mask` (returned if `'u_min'` is set) |
| `sensor_data.ux_rms` | rms of the time varying particle velocity in the x-direction recorded at the sensor positions given by `sensor.mask` (returned if `'u_rms'` is set) |
| `sensor_data.ux_final` | final particle velocity field in the x-direction at all grid points within the domain (returned if `'u_final'` is set) |
| `sensor_data.ux_max_all` | maximum particle velocity in the x-direction recorded at all grid points within the domain (returned if `'u_max_all'` is set) |
| `sensor_data.ux_min_all` | minimum particle velocity in the x-direction recorded at all grid points within the domain (returned if `'u_min_all'` is set) |
| `sensor_data.ux_non_staggered` | time varying particle velocity in the x-direction recorded at the sensor positions given by `sensor.mask` after shifting to the non-staggered grid (returned if `'u_non_staggered'` is set) |
| `sensor_data.Ix` | time varying acoustic intensity in the x-direction recorded at the sensor positions given by `sensor.mask` (returned if `'I'` is set) |
| `sensor_data.Ix_avg` | average acoustic intensity in the x-direction recorded at the sensor positions given by `sensor.mask` (returned if `'I_avg'` is set) |

## Examples

- Simulations In One Dimension
- Photoacoustic Waveforms in 1D, 2D and 3D
- Source Smoothing
- Filtering A Delta Function Input Signal
- Modelling Power Law Absorption
- Modelling Nonlinear Wave Propagation

## See Also

`kspaceFirstOrderAS, kspaceFirstOrder2D, kspaceFirstOrder2D, kWaveGrid`
